# Environmental and Health Risks Posed by Heavy Metal Contamination of Groundwater in the Sunan Coal Mine, China

**DOI:** 10.3390/toxics10070390

**Published:** 2022-07-12

**Authors:** Lijuan Wang, Yuezan Tao, Bin Su, Lijun Wang, Peigui Liu

**Affiliations:** 1 School of Civil and Hydraulic Engineering, Hefei University of Technology, Hefei 230009, China; taoyuezan@163.com (Y.T.); liupg2512@163.com (P.L.); 2 Department of Municipal and Transportation Engineering, Anhui Technical College of Water Resources and Hydroelectric Power, Hefei 231603, China; 3 Anhui and Huaihe Water Conservancy Science Research Institute, Hefei 230088, China; shubing198003@163.com; 4 Anhui Survey and Design Institute of Water Resources and Hydropower Company Limited, Hefei 230008, China; lijunwang1982@163.com

**Keywords:** groundwater, environmental geochemistry, heavy metals, environmental quality, human health risks

## Abstract

Groundwater is often used for domestic and irrigation purposes, even in mining areas. Mine drainage, rainfall, and infiltration cause heavy metal enrichment, adversely affecting the groundwater and harming human health. In this study, water samples (October 2021) in the Suzhou southern coal mining area were analyzed for the heavy metals As, Cr, Cu, Fe, Mn, Pb, and Zn to determine potential effects of heavy metal contamination on environmental quality and human health. It was found that 22% and 31% of the sampling sites had “excellent” and “good” water quality, respectively. Excessive concentrations of Fe and Mn were detected in 47% and 72% of the samples, respectively. The non-carcinogenic health risk values of As, Cr, Cu, Fe, Mn, Pb, and Zn were below the negligible levels of health risk set by various environmental agencies. Content ranking was as follows: Fe > Mn > Cr > Cu > Pb > Zn > As, with Fe accounting for 43%. All sampling points exceeded the maximum acceptable level of Cr recommended by the agencies. Chromium, the major carcinogenic factor in the study area, contributed to 95.45% of the total health risk. Therefore, the authorities in this region must closely monitor three heavy metal elements—Fe, Mn, and Cr.

## 1. Introduction

Most cities worldwide use groundwater as a source of drinking water because of its reliability and generally good quality. However, human activities such as mining affect groundwater in many cities. Mining not only forms a groundwater landing funnel but also pollutes aquifers, adversely affecting the groundwater environment and seriously endangering the health and safety of residents.

Heavy metal pollution has a serious impact on human health [1,2,3,4]. As and Mn constitute the majority of heavy metal pollutants in groundwater and have received ample attention in research over the past few decades in many countries around the world [5,6,7,8]. They have been intensively studied by scholars from Bangladesh, India, Pakistan, Vietnam, and China [9,10]. Many scholars believe that exposure to As causes critical health problems such as skin and bladder cancers [11,12,13]. CrIII and CrVI are the main forms of chromium in the environment; CrVI can result in an increase in tumors, and the International Agency for Research on Cancer listed it as a confirmed human carcinogen as early as 1990 [14]. In addition, excessive Pb content in the environment causes neurological [15,16] and other health issues, including kidney problems, hypertension, liver cirrhosis, and skin irritation [17,18]. In recent years, a growing number of scholars have studied environmental problems caused by Fe and Mn [19]. It has been established that groundwater containing high concentrations of Fe, Mn, Cu, and Zn can pose risks to human health [20,21,22,23].

Although coal is an important source of energy, hazardous elements may be released into the environment during coal exploration, washing, and other processes in mining areas [24,25,26,27]. In addition, agricultural production may lead to the discharge of wastewater carrying pesticides and fertilizers, contributing to groundwater pollution. The discharge of domestic sewage and industrial wastewater may also cause groundwater pollution [28]. Health Risk Assessment (HRA) is a quantitative description of the risk degree of environmental pollutants harmful to human health by linking health with environmental pollution and taking the risk degree as the evaluation standard [29]. In this study, Health Risk Assessment was used to objectively estimate the degree of impact from damage by heavy metals pollution in groundwater on human health [30]. The purpose of this evaluation is to provide scientific support for health risk management, domestic water safety, and water environment protection [31,32,33,34,35]. Studying the health risks of heavy metals pollution of groundwater in coal mining areas can find out whether the groundwater was severely contaminated by coal mining activities, and thus provide a basis for groundwater protection policies [4,36,37].

To enhance the understanding of groundwater quality in Sunan, this study determines the environmental quality and human health risks of seven heavy metals, As, Cr, Cu, Fe, Mn, Pb, and Zn. Furthermore, this study provides a theoretical basis for the scientific correlation, development, and utilization of groundwater resources in the mining area [38,39,40].

## 2. Materials and Methods

### 2.1. Research Area

**Figure 1:**
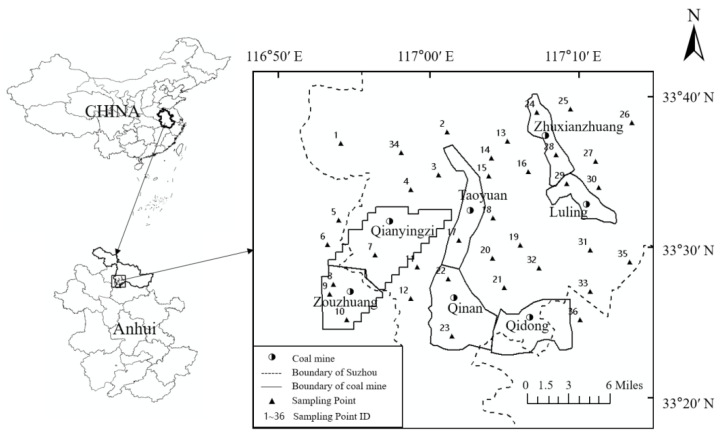
Sampling locations within of the study area.

The Sunan mining area (33°21′–33°42′ N, 116°45′–117°12′ E) is located in the middle of Huaibei Plain southeast of Suzhou City in China (Figure 1). The Sunan mining area is a vital coal production base in East China. Seven production mines, including Luling, Zhuxianzhuang, Qianyingzi, Zouzhuang, Taoyuan, Qinan, and Qidong, are situated in an area of approximately 450 km^2^. This area has a high population density and groundwater is used for both various human requirements and irrigation. In the process of coal mining, gangue, domestic wastewater, and agricultural non-point source pollution affect the hydrogeochemical characteristics of shallow groundwater; this can lead to organic or inorganic pollution and threaten groundwater quality and water supply security [41].

The groundwater aquifer systems in the mining area can be categorized as follows: loose pore, coal measure fractured, carbonate fractured, and interstitial aquifer systems. Water for human use comes from the loose rock pore water-bearing group. The lithology is mainly silty and secondary, and consists of fine sand, sub-sand, local fine sand, and silt. Three aquifer groups can be found in the vertical direction. The samples used in this study were primarily from the first aquifer, which has a depth of 100 m.

### 2.2. Sample Collection and Analysis

Samples were collected from 36 monitoring wells; these sampling points are civil wells or enterprise production wells, the distribution of which ensured that the entire study area was covered (Figure 1). The sampling wells are 7–50 m deep, and the water is characterized as phreatic. The measuring instrument used was the inductively coupled plasma mass spectrometer (ICP-MS) (NexlON 300, P.E. Corporation, Norwalk, CT, USA). Laboratory water was prepared for the Millipore ultrapure water preparation device. The mixed standard reserve solution comprised: 100 μg/mL (5%HNO_3_/tr Tartaric Acid/tr HF, American PE Corporation); the mass spectrometer tuning solution comprised: Be, Ce, Fe, In, Li, Mg, Pb, U (1 μg/L, 1%HNO_3_, P.E. Corporation, Norwalk, CT, USA); the mixed internal standard reserve solution comprised: 50 μg/mL Sc, 20 μg/mL Ge, 10 μg/mL In, Ir, Li, Rh, Tb, Y (5%HNO_3_/tr HCI, American PE Corporation); the Argon gas purity was above 99.99%; the nitric acid was superior grade pure.

For the sample preparation, 45.0 mL of the sample was measured accurately and put into the digestion tank, then 4.0 mL of concentrated nitric acid (GR) and 1.0 mL of concentrated hydrochloric acid (GR) were added, and placed in a microwave digestion instrument for microwave digestion at 170 °C for 10 min. The digestion solution was removed and cooled to room temperature, then moved to a 100 mL volumetric flask, filled to the scale with deionized water. This was shaken for testing. The Method Detection Limit (LD) for As, Cr, Cu, Fe, Mn, Pb, and Zn were 0.12 μg/L, 0.11 μg/L, 0.08 μg/L, 0.82 μg/L, 0.12 μg/L, 0.09 μg/L, and 0.67 μg/L, respectively. Each sample was measured 6 times in parallel, and the relative standard deviation was less than 4%; the standard added recovery rate was 95.0% to 107.4.1%; and the standard curve correlation coefficient (r) value was greater than 0.999. On the measurement set-up for Fe and As, He was selected as collision gas and collision reaction pool mode was adopted to eliminate the interference of ArO^+^ on Fe and ArCl^+^ on As. At the same time, Sc element was selected as the internal standard for the determination of Fe, and Ge was selected as the internal standard for the determination of As. The test results are shown in Table 1. The groundwater samples included in the study were collected following the norms and standards prescribed by the Ministry of Environmental Protection and the United States Environmental Protection Agency (US EPA) on sampling collection, transportation, storage, preparation, and instrumental analysis [42,43].

### 2.3. Groundwater Quality Index

A single factor index and a comprehensive index were used to evaluate the environmental quality of groundwater in the study area. The single factor evaluation compared the heavy metal concentration in each sample with the Class III division of the groundwater quality standards [44] which describes the quality required for centralized domestic and drinking water. Table 2 provides the parameters used for the classification of groundwater in China. The formula used to calculate the index is as follows:

(1) $$I_{i}=\frac{C_{i}}{C_{0}}$$

where *I_i_* is the single factor evaluation index, *C_i_* is the measured value of a given index concentration, and *C*_0_ is the upper limit concentration value of the water in the Class III division.

When *I_i_* ≤ 1, the water quality meets the corresponding water quality standard, and *I_i_* > 1, the water quality does not meet the selected water quality standard.

The comprehensive index was evaluated based on the Nemerow index method. The comprehensive index method was formulated as follows [44]:

(2) $$F=\sqrt{\frac{\left(F_{\max}^{2}+{\bar{F}}^{2}\right)}{2}}$$

where *F* is the Nemerow pollution index (Table 3); *F*_i_ is a single evaluation index; *F*_max_ is the maximum value in the score value of *F_i_*; $\bar{F}$ was obtained using Equation (3)

(3) $$\bar{F}=\frac{1}{n}\sum_{i=1}^{n}F_{i}$$

where *n* is the number of individual score values.

The comprehensive water quality evaluation grades corresponding to the Nemerow pollution index are shown in Table 3.

### 2.4. Human Health Risk

The health risk of carcinogens in drinking water was evaluated using the following equation [43]:

(4) $$R_{i}^{c}=\frac{1-\exp\left(-D_{i}q_{i}\right)}{L}$$

where $R_{i}^{c}$ is the average annual risk of carcinogenesis for one person caused by the carcinogen *i* in drinking water, a^−1^, *Di* is the mean exposure dose of carcinogen *i* in drinking water for a single person each day and was obtained using Equation (6), mg/(kg·d), *qi* is the carcinogenic potency factor of carcinogen *i* in drinking water, mg/(kg·d). A value of 41 for Cr and 15 for As [45] was used in the present study, and *L* is the average human lifespan, assumed to be 70 years.

The health risk of non-carcinogens in drinking water was assessed using the following equation:

(5) $$R_{i}^{n}=\frac{D_{i}\times 10^{-6}}{RfD_{i}\times L}$$

where $R_{i}^{n}$ is the average annual risk to individuals by non-carcinogens *i* in drinking water (a^−1^), $RfD_{i}$ is the reference dose of the non-carcinogen *i* in drinking water, mg/(kg·d). The values of $RfD_{i}$ are shown in Table 4.

The *D_i_* is calculated using Equation (6).

*D_i_* = (*CW* × *IR* × *EF* × *ED*)/(*BW* × *AT*) (6)

where *CW* is the concentration of the heavy metal in the groundwater, mg/L, *IR* is the daily intake of drinking water (a value of 2.2 L/d was used), *EF* is the exposure frequency (the value used was 365 days/year), *ED* is the exposure duration (70 years for the carcinogen, and 30 years for the non-carcinogen), *BW* is the body weight in kg (70 kg was selected for the adults in Suzhou City), *AT* is the average exposure time (days), which was calculated as 365 × *ED*.

The total annual risk of carcinogenesis caused by the carcinogens in groundwater was calculated using Equation (7). The reference values used for each risk level were provided by the international standards of various agencies, as shown in Table 5.

(7) $$R=\sum_{i=1}^{n}R_{i}^{c}+\sum_{i=1}^{n}R_{i}^{n}$$

## 3. Results and Discussion

### 3.1. Environmental Quality Research

#### 3.1.1. Single-Factor Evaluation

**Figure 2:**
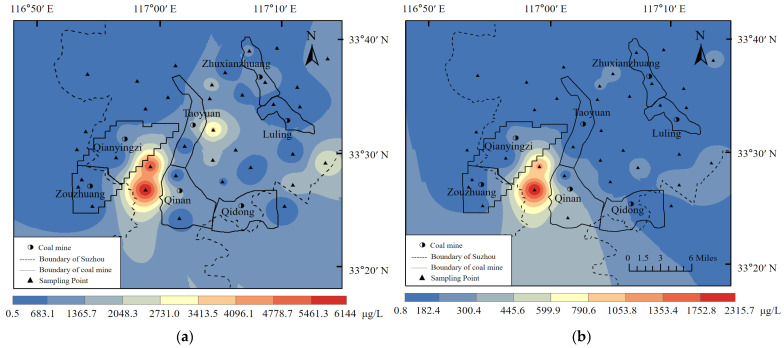
Concentration distribution. (**a**) Diagram of Fe concentration distribution (**b**) Diagram of Mn concentration distribution.

Analysis of the heavy metal contents at the 36 sampling sites revealed that the seven heavy metal contents investigated could be graded as Fe > Mn > Zn > Cr > Cu > As > Pb (Table 6). Furthermore, according to the analysis of the coefficient of variation of each heavy metal element at the sampling point, the coefficient of variation exceeded 1 (Table 6), and the hydro-chemical properties of groundwater showed great variability in space. After comparing the content of the seven heavy metals at each sampling point with the class III water quality standard of standard for groundwater quality for China (see Table 7), it was found that the water quality of 17 sampling points did not meet the Fe concentration requirements of the national water standard, accounting for 47%, and 26 sampling points did not meet the Mn concentration requirements of the national water standard, accounting for 72%. Moreover, the concentration of Fe and Mn at 15 sampling sites was excessive. The maximum excess multiples of Fe and Mn are 20.5 and 23.2, respectively, indicating a substantial excess. Of the 36 sampling sites, only six (17%) met the national water quality standards. The results showed that the groundwater in the area was contaminated with Fe and Mn. The concentration distribution map of iron and manganese is shown in Figure 2. Notably, the iron and manganese content was very high in some sampling points, which can likely be attributed to the seriously polluted well water here.

Water quality: the class III water quality standard of standard for groundwater quality, China.

The Sunan area is rich in coal, with total mining area covering 450 km^2^. Studies have shown that coal mining activities transform groundwater from a reducing environment to an oxidizing environment, promote the oxidation process of pyrite, and increase the iron content in groundwater through leaching. In addition, due to the large area of collapse pond formed by mining activities, Fe and Mn have penetrated the first aquifer under the influence of precipitation [46]. Some scholars have also found excessive Fe and Mn in groundwater in the mining cities of southern China, suggesting that pollution from Fe and Mn in the Sunan mining area may be responsible [47,48,49].

#### 3.1.2. Comprehensive Evaluation

The results of scoring the water quality of each sample site are shown in Table 8. Among the 36 sampling sites, eight had excellent water quality, 11 were good, 13 were poor, and four were very poor, accounting for 22%, 31%, 36%, and 11%, respectively. The good water quality and exceptional water quality in the study area accounted for 53%, indicating that water quality in the study area was poor and that nearly half of the wells were polluted. These results verify the above single-factor evaluation findings and may be caused by human activities (such as mining and over-exploitation of underground water) contaminating the groundwater in this area.

### 3.2. Human Health Risk Analysis

#### 3.2.1. Non-Carcinogenic Health Risk

**Figure 3:**
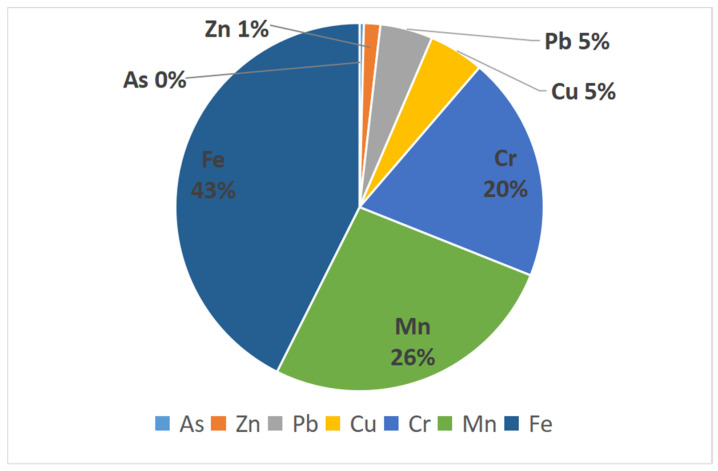
Non-carcinogenic health risk ratios.

The reference value of the risk level recommended by some agencies is tabulated in Table 5. The calculated non-carcinogenic health risk values for the seven heavy metal elements, As, Cr, Cu, Fe, Mn, Pb, and Zn, are shown in the table, with a maximum value of 9.20 × 10^−9^ (Table 9). The total non-carcinogenic health risks are well below the negligible level stipulated by the agencies (Table 5). The results show that the level of non-carcinogenic health risk caused by heavy metals in shallow groundwater is low and does not cause significant harm to humans. After calculations of non-carcinogenic health risk values, it has been found that the contribution of the seven heavy metals to the non-carcinogenic health risk was in the order of Fe > Mn > Cr > Cu > Pb > Zn > As. Among them, Fe and Mn contributed the most, accounting for 43% and 26% of the total risk values, respectively (Figure 3).

Iron and Mn are common heavy metal elements in groundwater. Manganese is one of the essential trace elements in the human body [34]. Iron, also a trace element, is beneficial to human health [50]. The use of water with iron and manganese levels exceeding the standard, however, would harm economic production and human health. Drinking water guidelines developed by the World Health Organization require iron and manganese levels to be no more than 0.3 mg/L and 0.4 mg/L, respectively [51]. To protect human health, the relevant Chinese authorities have formulated standards to regulate the content of Fe and Mn in water. China’s drinking water sanitation standards and Chinese groundwater quality standards stipulate that Fe in drinking water should be no more than 0.3 mg/L, and the limit for Mn is 0.1 mg/L. Studies have shown that long-term exposure to excessive iron and manganese cause non-carcinogenic health risks. These risks include Parkinson’s disease, cardiovascular disease, hyperkeratosis, diabetes, altered pigmentation, Alzheimer’s disease, kidney, liver, respiratory, and neurological disorders [40,52,53,54].

The exceeded values for iron and manganese reported in this study are mainly attributable to anthropogenic sources, e.g., agricultural, chemical industry, and coal mining waste, with a small amount originating from geological origin processes such as the weathering of bedrock materials (feldspar and evaporite) in the groundwater system [55,56]. Similar findings have been made across Asia. Excessive iron and manganese value have been reported in the Basundhara coal mining region [57], coal mines in Pakistan [58], a typical mining area in Northern Anhui Province, China [59], and a coal mine area in the Ordos basin, north of the Chinese Loess Plateau [60]. The growing body of evidence can provide insight into the cause of groundwater pollution, and help to curb groundwater pollution.

#### 3.2.2. Carcinogenic Health Risks

**Figure 4:**
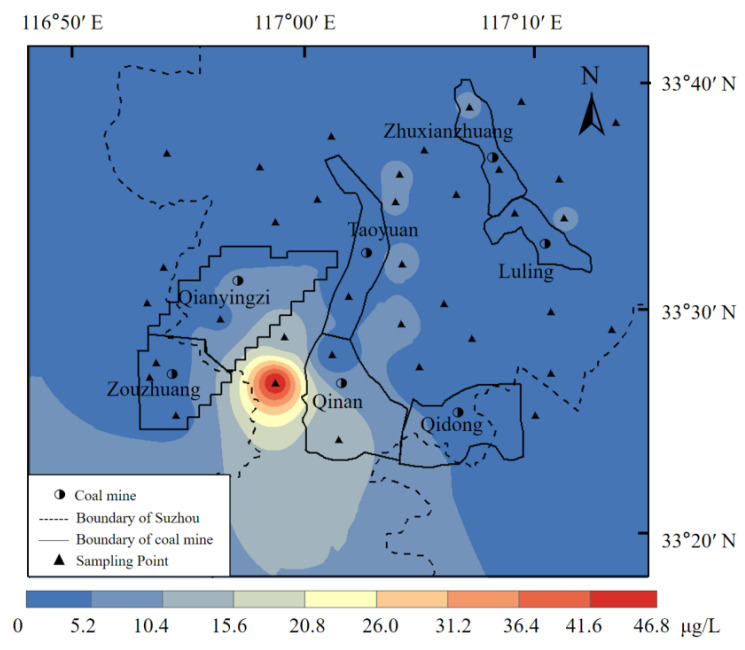
Diagram of Cr concentration distribution.

The carcinogenic risk calculations are shown in Table 9. In addition to two samples, the carcinogenic risk value for Cr was between 1.84 × 10^−6^ and 8.37 × 10^−4^. All samples exceeded the maximum acceptable Cr level recommended by the Swedish Environmental Protection Agency, the Dutch Ministry of Construction and Environment, and the Royal Society. Seventeen sites (47%) exceeded the International Commission on Radiological Protection (ICRP)-recommended maximum acceptable cancer-causing risk levels, and nine sites (25%) exceeded the maximum acceptable level recommended by the US EPA. The carcinogenic risk for As was between 6.73 × 10^−7^ and 1.68 × 10^−5^, exceeding the maximum acceptable levels recommended by the Swedish Environmental Protection Agency, the Dutch Ministry of Construction and Environment, and the Royal Society, but is below the maximum acceptable level recommended by the ICRP and US EPA. The overall cancer risk caused by As and Cr exceeded the maximum acceptable level recommended by the Swedish Environmental Protection Agency, the Dutch Ministry of Construction and Environment, and the Royal Society. Therefore, the relevant authorities should seriously consider the carcinogenic risk of heavy metals in shallow groundwater in the study area. When comparing As and Cr, the carcinogenic risk value for As was generally at 10^−6^, whereas that for Cr was between 10^−4^ and 10^−5^. The calculated carcinogenic risk of As was 4.55% of the total cancer risk, whereas Cr accounted for 95.45% of the total cancer risk. Thus, the carcinogenic risk of Cr was much higher than that of As, and Cr was the major carcinogenic heavy metal element in this region. The concentration distribution map of Cr is shown in Figure 4.

Chromium is widely found in groundwater [61], and CrIII and CrVI are the primary forms of Cr in the environment, with different toxicities [62,63]. Notably, CrIII is one of the essential trace elements in the human body and is involved in the metabolism of human blood glucose and three nutrients. It can increase insulin activity and reduce the risk of diabetes. However, excessive CrIII may have led to long-term toxicity and carcinogenicity [64]. CrVI is 100 times more toxic than CrVI; it is highly carcinogenic and allergic, and it is listed as one of the eight chemicals causing the greatest harm to the human body. The International Institute for Research on Cancer defines it as a human class 1 carcinogen (a carcinogenic chemical for humans) [65]. CrVI can cause genomic DNA damage and the oxidative deterioration of blood lipids and proteins, damaging the immune, nervous, and reproductive systems and kidney function [66]. Considering the health risks generated by Cr in the Sunan mining area, the local government should formulate effective measures to strengthen protection.

The high concentration of Cr can be attributed to artificial sources. High Cr concentrations may be caused by the leaching of coal wastewater from the nearby coal industry, or by agricultural irrigation that causes the infiltration of Cr from pesticides and fertilizers into the groundwater [67]. In this study, several points had higher Cr concentrations, but as seen from the concentration plot, these points were prevalent in the study area and were not related to their proximity to the coal mine. At one of the points, Cr had a particularly high concentration, possibly because the well was heavily contaminated. It is certain that the groundwater in these wells is disturbed by anthropogenic activities, and the concentration of Cr in the well water varies due to the distribution of the formation’s cracks and soil quality. There have also been many reports of excessive Cr concentrations in coal mine production areas in other countries. Mean Cr values of 3.39 mg/L [68], 44.6 mg/L [69], and 19.756 mg/L [70] were reported in the coal basins of northwest Bangladesh, Raniganj, India, and Thrace, Turkey, respectively. These studies sufficiently demonstrate that the high Cr concentration correlates with coal mining, and standardized coal mining is needed to reduce groundwater interference by human activities.

#### 3.2.3. Total Health Risk

As shown in Table 9, the total carcinogenic health risk was between 1.85 × 10^−6^ to 8.54 × 10^−4^, but the non-carcinogenic risk was generally between 10^−11^ to 10^−9^, indicating that carcinogenic risk was the primary contributor to total health risk. The mean carcinogenic risk of the 36 sampling points was 8.14 × 10^−5^, amounting to 99.99% of the total health risk. The results showed that the health risks of shallow groundwater in the area consisted almost entirely of carcinogenic risks. Chromium contributes 95.45% of the risk; therefore, groundwater Cr in the region requires special attention from the local government.

## 4. Conclusions

In this study, 36 groundwater samples were collected in the Sunan mining area in China; As, Cr, Cu, Fe, Mn, Pb, and Zn were tested by ICP-MS analysis to determine the environmental quality and the effects on human health.

The results of the environmental quality study showed a rank order of Fe > Mn > Zn > Cr > Cu > As > Pb with a large concentration coefficient of variation. Seventeen percent of the sampling sites met the water quality standards for centralized drinking water sources, industrial, and agricultural water, whereas 47% and 72% of the sampling sites exceeded the stipulated Fe and Mn concentrations, respectively. Sampling sites with excellent, good, poor, and extremely poor water quality grades accounted for 22%, 31%, 36%, and 11%, respectively. The human health risk study showed that non-carcinogenic health risk values were lower than the negligible level given by various agencies. Elements were ranked according to their non-carcinogenic health risk value as Fe > Mn > Cr > Cu > Pb > Zn > As, of which Fe and Mn accounted for 43% and 26%, respectively. All the carcinogenic risk values exceeded the agency-recommended maximum acceptable level. Chromium was the major carcinogenic factor in the study area and contributed 95.45% of the total health risk. Therefore, special attention should be given to Fe, Mn, and Cr elements in groundwater in this area. The results of this study will provide environmental researchers with further insights into trends in heavy metal pollution in over-extracted groundwater areas. We quantified the health risks related to a range of common groundwater pollutants, and these results will assist in developing effective countermeasures for groundwater pollution.

## Figures and Tables

**Table 1:** Heavy metal concentrations in groundwater (μg/L).

Sampling Point	Well Depth (m)	Cr	Cu	Fe	Mn	Pb	Zn	As
1	36.7	3.70	1.82	13.00	154	0.52	11.03	0.80
2	32.0	2.30	0.13	38.73	149	0.25	8.83	0.50
3	30.7	0.20	0.27	121	61.88	<LD	3.69	0.90
4	33.0	0.40	<LD	15.92	0.50	0.23	12.92	0.80
5	45.0	1.40	0.37	3.40	5.60	0.48	8.98	0.50
6	40.0	3.92	3.52	<LD	221	0.60	6.95	<LD
7	33.0	4.33	0.60	2.10	9.00	0.44	7.35	0.40
8	30.0	0.30	0.36	176	202	0.17	8.33	0.90
9	15.0	3.57	1.22	69.66	6.49	<LD	8.84	0.50
10	40.0	3.81	0.68	199	228	0.19	4.83	<LD
11	9.0	12.53	21.38	5324	1600	<LD	56.85	<LD
12	45.1	46.88	1.48	6150	2318	<LD	29.45	2.50
13	47.9	<LD	11.77	273	320	<LD	31.63	1.10
14	27.0	6.70	0.44	1752	282	0.60	<LD	<LD
15	25.0	5.68	0.54	1113	186	<LD	499.30	0.70
16	6.2	3.20	1.05	409	137	<LD	10.47	0.80
17	10.0	2.10	0.16	135	39.29	0.20	12.25	0.40
18	7.6	6.33	0.68	3500	130	0.19	34.36	0.90
19	18.0	0.20	0.23	1335	252	0.21	<LD	0.40
20	23.5	8.73	0.53	1453	214	1.00	5.67	0.40
21	20.0	2.10	1.55	635	157	2.07	12.45	<LD
22	12.5	0.30	0.87	222	174	0.53	11.96	<LD
23	10.0	12.12	1.82	184	528	<LD	3.74	0.80
24	9.0	5.94	0.13	706	85.79	<LD	17.02	1.00
25	23.5	1.20	0.27	4.50	37.46	0.50	42.52	0.60
26	47.3	0.50	1.44	1328	290	<LD	174.50	0.60
27	24.2	0.80	1.14	414	105	<LD	10.19	0.90
28	45.0	3.55	1.61	1353	111	6.63	7.47	<LD
29	32.0	0.30	0.43	93.13	3.20	<LD	1.51	0.40
30	43.0	6.32	1.06	315	13.45	0.16	5.17	0.50
31	9.8	0.30	0.83	270	160	0.26	3.36	0.30
32	16.8	<LD	0.81	75.94	294	0.86	2.43	0.40
33	25.0	<LD	1.01	1887	383	<LD	5.16	0.80
34	18.2	3.62	1.96	674	181	0.17	12.45	0.50
35	32.2	<LD	0.59	2689	346	0.20	34.30	<LD
36	48.0	0.30	0.25	255	195	0.22	16.53	0.40

**Table 2:** Classification of groundwater by quality.

Order Number	Class	Scope of Application
1	I class	Various uses
2	II class	Various uses
3	III class	Centralized domestic and drinking water sources, industrial and agricultural water use
4	IV class	Used for agriculture and in the industry, but can be used for drinking after appropriate treatment
5	V class	Choose according to the purpose of use

**Table 3:** Groundwater quality classification standard and single-component scoring standard.

Water Quality Classification	Individual Component Score Values $F_{i}$	The Nemerow Pollution Index, *F*	Water Quality Classification
I	0	<0.80	Excellent
II	1	0.80–2.50	Good
III	3	2.50–4.25	Better
IV	6	4.25–7.20	Poor
V	10	>7.20	Extremely Poor

**Table 4:** Reference dose of target substance of non-chemical carcinogens (*RfD*) (mg/kg/d).

Target Substance	*RfD Value*
**Cr**	3.0 × 10^−3^
**Cu**	5 × 10^−3^
**Fe**	0.3
**Mn**	1.4 × 10^−1^
**Pb**	1.4 × 10^−3^
**Zn**	3.0 × 10^−1^
**As**	0.02

**Table 5:** Reference values of the risk level (a^−1^).

Institution Name	Maximum Acceptable Level	Negligible Level	Remarks
The Swedish Environmental Protection Agency	1.0 × 10^−6^	-	Chemical pollutant
The Dutch Ministry of Construction and the Environment	1.0 × 10^−6^	1.0 × 10^−8^	Chemical pollutant
Royal Society	1.0 × 10^−6^	1.0 × 10^−7^	-
International Commission on Radiological Protection	5.0 × 10^−5^	-	Radiation
US Environmental Protection Agency	1.0 × 10^−4^	-	-

**Table 6:** List of groundwater heavy metal elements’ statistical characteristics (μg/L).

Index	Cr	Cu	Fe	Mn	Pb	Zn	As
Max	46.88	21.38	6150	2318	6.63	499.30	2.50
Min	<LD	<LD	<LD	0.50	<LD	<LD	<LD
Mean	4.27	1.75	922	266	0.47	31.20	0.55
Standard deviation	8.01	3.89	1440	442	1.13	85.57	0.47
Coefficient of variation (100%)	1.87	2.22	1.56	1.66	2.43	2.74	0.85

**Table 7:** Terms of the groundwater sample in the study area exceeding the III water standard.

Index	Cr	Cu	Fe	Mn	Pb	Zn	As
Indices limit of groundwater quality classification (mg/L) [44]	≤0.05	≤1.00	≤0.3	≤0.10	≤0.01	≤1.00	≤0.01
Excess sampling point (Each)	0	0	17	26	0	0	0
Exceeding the standard (%)	0	0	47	72	0	0	0
Maximum excess multiple	0.00	0.00	20.50	23.20	0.00	0.00	0.00

**Table 8:** The calculation of *F* value.

Sample Point	*F* (Cr)	*F* (Cu)	*F* (Fe)	*F* (Mn)	*F* (Pb)	*F* (Zn)	*F* (As)	$F_{\max}$	$\bar{F}$	*F*
1	0	0	0	3	0	0	0	3	0.75	2.19
2	0	0	0	3	0	0	0	3	0.75	2.19
3	0	0	1	1	0	0	0	1	0.38	0.76
4	0	0	0	0	0	0	0	0	0.00	0.00
5	0	0	0	0	0	0	0	0	0.00	0.00
6	0	0	0	3	0	0	0	3	0.75	2.19
7	0	0	0	0	0	0	0	0	0.00	0.00
8	0	0	1	3	0	0	0	3	0.88	2.21
9	0	0	0	0	0	0	0	0	0.00	0.00
10	0	0	1	3	0	0	0	3	0.88	2.21
11	3	1	10	10	0	1	0	10	4.38	7.72
12	3	0	10	10	0	0	1	10	4.25	7.68
13	0	1	3	3	0	0	1	3	1.38	2.33
14	1	0	6	3	0	0	0	6	2.00	4.47
15	1	0	6	3	0	1	0	6	2.13	4.50
16	0	0	6	3	0	0	0	6	1.88	4.44
17	0	0	1	0	0	0	0	1	0.25	0.73
18	1	0	10	3	0	0	0	10	3.00	7.38
19	0	0	6	3	0	0	0	6	1.88	4.44
20	1	0	6	3	0	0	0	6	2.00	4.47
21	0	0	6	3	0	0	0	6	1.88	4.44
22	0	0	3	3	0	0	0	3	1.13	2.27
23	3	0	1	3	0	0	0	3	1.25	2.30
24	1	0	6	1	0	0	1	6	1.88	4.44
25	0	0	0	0	0	0	0	0	0.00	0.00
26	0	0	6	3	0	1	0	6	2.00	4.47
27	0	0	6	3	0	0	0	6	1.88	4.44
28	0	0	6	3	3	0	0	6	2.25	4.53
29	0	0	0	0	0	0	0	0	0.00	0.00
30	1	0	6	0	0	0	0	6	1.63	4.40
31	0	0	3	3	0	0	0	3	1.13	2.27
32	0	0	0	3	0	0	0	3	0.75	2.19
33	0	0	6	3	0	0	0	6	1.88	4.44
34	0	0	6	3	0	0	0	6	1.88	4.44
35	0	0	10	3	0	0	0	10	2.88	7.36
36	0	0	3	3	0	0	0	3	1.13	2.27

**Table 9:** Health risk (a^−1^).

Sample Point	Non-Carcinogenic Risk							Carcinogenic Risk		Total Health Risk
	Cr	Cu	Fe	Mn	Pb	Zn	As	Cr	As	
1	5.54 × 10^−10^	1.63 × 10^−10^	1.95 × 10^−11^	4.95 × 10^−10^	1.67 × 10^−10^	1.65 × 10^−11^	1.80 × 10^−11^	6.79 × 10^−5^	5.39 × 10^−6^	7.33 × 10^−5^
2	3.44 × 10^−10^	1.17 × 10^−11^	5.80 × 10^−11^	4.77 × 10^−10^	8.02 × 10^−11^	1.32 × 10^−11^	1.12 × 10^−11^	4.23 × 10^−5^	3.37 × 10^−6^	4.56 × 10^−5^
3	2.99 × 10^−11^	2.42 × 10^−11^	1.81 × 10^−10^	1.98 × 10^−10^	0	5.52 × 10^−12^	2.02 × 10^−11^	3.68 × 10^−6^	6.06 × 10^−6^	9.74 × 10^−6^
4	5.99 × 10^−11^	0	2.38 × 10^−11^	1.60 × 10^−12^	7.38 × 10^−11^	1.93 × 10^−11^	1.80 × 10^−11^	7.36 × 10^−6^	5.39 × 10^−6^	1.27 × 10^−5^
5	2.10 × 10^−10^	3.35 × 10^−11^	5.09 × 10^−12^	1.80 × 10^−11^	1.54 × 10^−10^	1.34 × 10^−11^	1.12 × 10^−11^	2.57 × 10^−5^	3.37 × 10^−6^	2.91 × 10^−5^
6	5.86 × 10^−10^	3.16 × 10^−10^	4.49 × 10^−13^	7.09 × 10^−10^	1.92 × 10^−10^	1.04 × 10^−11^	0	7.19 × 10^−5^	0	7.19 × 10^−5^
7	6.49 × 10^−10^	5.40 × 10^−11^	3.14 × 10^−12^	2.89 × 10^−11^	1.41 × 10^−10^	1.10 × 10^−11^	8.98 × 10^−12^	7.96 × 10^−5^	2.69 × 10^−6^	8.23 × 10^−5^
8	4.49 × 10^−11^	3.20 × 10^−11^	2.63 × 10^−10^	6.47 × 10^−10^	5.45 × 10^−11^	1.25 × 10^−11^	2.02 × 10^−11^	5.52 × 10^−6^	6.06 × 10^−6^	1.16 × 10^−5^
9	5.34 × 10^−10^	1.09 × 10^−10^	1.04 × 10^−10^	2.08 × 10^−11^	0	1.32 × 10^−11^	1.12 × 10^−11^	6.56 × 10^−5^	3.37 × 10^−6^	6.89 × 10^−5^
10	5.70 × 10^−10^	6.12 × 10^−11^	2.97 × 10^−10^	7.32 × 10^−10^	6.09 × 10^−11^	7.23 × 10^−12^	0	6.99 × 10^−5^	0	6.99 × 10^−5^
11	1.88 × 10^−9^	1.92 × 10^−9^	7.97 × 10^−9^	5.13 × 10^−9^	0	8.51 × 10^−11^	0	2.29 × 10^−4^	0	2.29 × 10^−4^
12	7.02 × 10^−9^	1.33 × 10^−10^	9.20 × 10^−9^	7.43 × 10^−9^	2.89 × 10^−11^	4.41 × 10^−11^	5.61 × 10^−11^	8.37 × 10^−4^	1.68 × 10^−5^	8.54 × 10^−4^
13	1.50 × 10^−11^	1.06 × 10^−9^	4.09 × 10^−10^	1.02 × 10^−9^	0	4.73 × 10^−11^	2.47 × 10^−11^	1.84 × 10^−6^	7.41 × 10^−6^	9.25 × 10^−6^
14	1.00 × 10^−9^	3.95 × 10^−11^	2.62 × 10^−9^	9.05 × 10^−10^	1.92 × 10^−10^	3.20 × 10^−13^	0	1.23 × 10^−4^	0	1.23 × 10^−4^
15	8.50 × 10^−10^	4.85 × 10^−11^	1.67 × 10^−9^	5.97 × 10^−10^	0	7.47 × 10^−10^	1.57 × 10^−11^	1.04 × 10^−4^	4.71 × 10^−6^	1.09 × 10^−4^
16	4.79 × 10^−10^	9.43 × 10^−11^	6.13 × 10^−10^	4.39 × 10^−10^	0	1.57 × 10^−11^	1.80 × 10^−11^	5.88 × 10^−5^	5.39 × 10^−6^	6.42 × 10^−5^
17	3.14 × 10^−10^	1.44 × 10^−11^	2.02 × 10^−10^	1.26 × 10^−10^	6.38 × 10^−11^	1.83 × 10^−11^	8.98 × 10^−12^	3.86 × 10^−5^	2.69 × 10^−6^	4.13 × 10^−5^
18	9.47 × 10^−10^	6.11 × 10^−11^	5.24 × 10^−9^	4.18 × 10^−10^	6.16 × 10^−11^	5.14 × 10^−11^	2.02 × 10^−11^	1.16 × 10^−4^	6.06 × 10^−6^	1.22 × 10^−4^
19	2.99 × 10^−11^	2.07 × 10^−11^	2.00 × 10^−9^	8.09 × 10^−10^	6.67 × 10^−11^	6.23 × 10^−13^	8.98 × 10^−12^	3.68 × 10^−6^	2.69 × 10^−6^	6.38 × 10^−6^
20	1.31 × 10^−9^	4.76 × 10^−11^	2.17 × 10^−9^	6.85 × 10^−10^	3.21 × 10^−10^	8.48 × 10^−12^	8.98 × 10^−12^	1.60 × 10^−4^	2.69 × 10^−6^	1.63 × 10^−4^
21	3.14 × 10^−10^	1.39 × 10^−10^	9.50 × 10^−10^	5.05 × 10^−10^	6.64 × 10^−10^	1.86 × 10^−11^	0	3.86 × 10^−5^	0	3.86 × 10^−5^
22	4.49 × 10^−11^	7.81 × 10^−11^	3.32 × 10^−10^	5.58 × 10^−10^	1.71 × 10^−10^	1.79 × 10^−11^	0	5.52 × 10^−6^	0	5.52 × 10^−6^
23	1.81 × 10^−9^	1.63 × 10^−10^	2.76 × 10^−10^	1.69 × 10^−9^	0	5.60 × 10^−12^	1.80 × 10^−11^	2.21 × 10^−4^	5.39 × 10^−6^	2.27 × 10^−4^
24	8.89 × 10^−10^	1.17 × 10^−11^	1.06 × 10^−9^	2.75 × 10^−10^	0	2.55 × 10^−11^	2.24 × 10^−11^	1.09 × 10^−4^	6.73 × 10^−6^	1.16 × 10^−4^
25	1.80 × 10^−10^	2.42 × 10^−11^	6.73 × 10^−12^	1.20 × 10^−10^	1.60 × 10^−10^	6.36 × 10^−11^	1.35 × 10^−11^	2.21 × 10^−5^	4.04 × 10^−6^	2.61 × 10^−5^
26	7.48 × 10^−11^	1.29 × 10^−10^	1.99 × 10^−9^	9.31 × 10^−10^	0	2.61 × 10^−10^	1.35 × 10^−11^	9.20 × 10^−6^	4.04 × 10^−6^	1.32 × 10^−5^
27	1.20 × 10^−10^	1.03 × 10^−10^	6.20 × 10^−10^	3.38 × 10^−10^	0	1.53 × 10^−11^	2.02 × 10^−11^	1.47 × 10^−5^	6.06 × 10^−6^	2.08 × 10^−5^
28	5.32 × 10^−10^	1.45 × 10^−10^	2.02 × 10^−9^	3.57 × 10^−10^	2.13 × 10^−9^	1.12 × 10^−11^	2.24 × 10^−12^	6.52 × 10^−5^	6.73 × 10^−7^	6.59 × 10^−5^
29	4.49 × 10^−11^	3.85 × 10^−11^	1.39 × 10^−10^	1.03 × 10^−11^	0	2.25 × 10^−12^	8.98 × 10^−12^	5.52 × 10^−6^	2.69 × 10^−6^	8.22 × 10^−6^
30	9.46 × 10^−10^	9.48 × 10^−11^	4.72 × 10^−10^	4.31 × 10^−11^	5.10 × 10^−11^	7.74 × 10^−12^	1.12 × 10^−11^	1.16 × 10^−4^	3.37 × 10^−6^	1.19 × 10^−4^
31	4.49 × 10^−11^	7.41 × 10^−11^	4.04 × 10^−10^	5.13 × 10^−10^	8.21 × 10^−11^	5.03 × 10^−12^	6.73 × 10^−12^	5.52 × 10^−6^	2.02 × 10^−6^	7.54 × 10^−6^
32	0	7.30 × 10^−11^	1.14 × 10^−10^	9.42 × 10^−10^	2.74 × 10^−10^	3.64 × 10^−12^	8.98 × 10^−12^	0	2.69 × 10^−6^	2.70 × 10^−6^
33	0	9.10 × 10^−11^	2.82 × 10^−9^	1.23 × 10^−9^	0	7.73 × 10^−12^	1.80 × 10^−11^	0	5.39 × 10^−6^	5.39 × 10^−6^
34	5.42 × 10^−10^	1.76 × 10^−10^	1.01 × 10^−9^	5.82 × 10^−10^	5.45 × 10^−11^	1.86 × 10^−11^	1.12 × 10^−11^	6.65 × 10^−5^	3.37 × 10^−6^	6.99 × 10^−5^
35	1.50 × 10^−11^	5.28 × 10^−11^	4.02 × 10^−9^	1.11 × 10^−9^	6.54 × 10^−11^	5.13 × 10^−11^	0	1.84 × 10^−6^	0	1.85 × 10^−6^
36	4.49 × 10^−11^	2.22 × 10^−11^	3.82 × 10^−10^	6.24 × 10^−10^	6.93 × 10^−11^	2.47 × 10^−11^	8.98 × 10^−12^	5.52 × 10^−6^	2.69 × 10^−6^	8.22 × 10^−6^
Mean	6.40 × 10^−10^	1.57 × 10^−10^	1.38 × 10^−9^	8.53 × 10^−10^	1.49 × 10^−10^	4.67 × 10^−11^	1.23 × 10^−11^	7.77 × 10^−5^	3.70 × 10^−6^	8.14 × 10^−5^

## Data Availability

Data supporting the results during the current study are available from the corresponding author on reasonable request and all data provided in the manuscript.
